# Neoadjuvant lenvatinib for inoperable thyroid cancer: A case report and literature review

**DOI:** 10.1002/cnr2.1466

**Published:** 2021-06-08

**Authors:** Khalid Alshehri, Yousuf Alqurashi, Mazin Merdad, Shaza Samargandy, Razan Daghistani, Hani Marzouki

**Affiliations:** ^1^ Department of Otorhinolaryngology King Abdulaziz University Hospital Jeddah Saudi Arabia; ^2^ Endocrine Unite King Abdulaziz University Hospital Jeddah Saudi Arabia; ^3^ Department of Radiology King Abdulaziz University Hospital Jeddah Saudi Arabia

**Keywords:** lenvatinib, neoadjuvant, thyroid cancer, tyrosine kinase inhibitors

## Abstract

**Background:**

Poorly differentiated thyroid cancer (PDTC) is now classified as a separate thyroid tumor entity. It has male predominance and poor prognosis compared to differentiated TC.

**Case:**

We report a case of a patient with PDTC who was previously deemed inoperable. A trial of neoadjuvant lenvatinib therapy was given to the patient after that the tumor become operable and the surgery went successfully.

**Conclusions:**

Lenvatinib is a feasible option in patients with inoperable TC and can stabilize the lesion size or even reduce it, leading to a more favorable surgical outcome.

## 1 INTRODUCTION

Thyroid cancer (TC) is a common malignancy in over 50 000 newly diagnosed patients annually in the United States alone.^1^ Globally, the most common histological subtype of TC is papillary TC, followed by follicular TC.^2^ Other TC subtypes are relatively rare.^2^ Poorly differentiated TC (PDTC) was historically considered a tumor with intermediate characteristics between differentiated TC and anaplastic TC, and in 2004, it was classified as a separate thyroid tumor entity.^3^, ^4^ It has male predominance and poor prognosis compared to differentiated TC. Patients with PDTC tend to be older in age, with advanced locoregional or metastatic disease at presentation.^5^ In these cases, the decision of surgery as an initial management can be challenging due to potential morbidity without achieving complete resection.^6^

A limited number of case reports have investigated the role of tyrosine kinase inhibitors (TKIs) in PDTC or differentiated TC as neoadjuvant therapy for inoperable TC in order to facilitate complete resection with subsequent surgery. Lenvatinib is an oral TKI approved by the US Food and Drug Administration for radioactive iodine (RAI)‐refractory TC.^7^ In 2016, the landmark SELECT trial concluded that lenvatinib demonstrates significant response rates and progression‐free survival in the treatment of RAI‐refractory progressive TC and should be considered in the setting of clinically relevant or symptomatic disease progression.^8^

Herein, we report a case of a patient with PDTC who was previously deemed inoperable but was later successfully operated on with complete excision after a trial of neoadjuvant lenvatinib therapy. We also present a series of cases from the literature that utilized neoadjuvant TKIs in the setting of inoperable TC.

## 2 CASE REPORT

The patient was a 56‐year‐old woman with diabetes mellitus, hypertension, and a history of right breast cancer treated with right mastectomy, six cycles of chemotherapy, and three sessions of external beam radiation therapy (EBRT). She had been in remission since 2015. In May 2018, she presented to the otorhinolaryngology clinic after noticing a midline neck mass that gradually increased in size over the course of 6 months. Initial workup yielded normal serum thyroid‐stimulating hormone levels and a large right thyroid mass with an extrathyroidal extension (ETE) seen on neck ultrasound. Computed tomography (CT) with intravenous contrast showed a large heterogeneous mass arising from the right thyroid lobe measuring 6.7 × 8.7 × 8.9 cm with multiple areas of calcification and necrosis. The lesion invaded the right internal jugular vein, and there was minimal nodularity of the surface of the right tracheal wall concerning invasion. There was no fat plane between this mass and the esophageal right lateral wall; hence, esophageal invasion could not be excluded. The right brachiocephalic vein was significantly compressed by the mass. Contrast‐enhanced CT of the chest also demonstrated multiple lung metastases without mediastinal or hilar lymphadenopathy and a solitary sternal osseous metastatic deposit with focal cortical erosion posteriorly.

Due to the history of breast cancer, she underwent a fluorodeoxyglucose‐positron emission tomography scan that did not show uptake at either the lung nodules or the sternal lesion. Therefore, the metastatic spots were more likely to originate from TC rather than breast cancer. The patient had an open thyroid true cut biopsy, which was consistent with PDTC. After thorough assessment, it was concluded that complete surgical resection of the tumor was unlikely. The patient's case was presented to the Head & Neck Tumor Board, and the consensus was to initially treat the patient palliatively with 15 sessions of 40 Gray EBRT as a means of local control, followed by chemotherapy or targeted therapy. A genomic profiling test from the thyroid sample indicated *HRAS‐BCORL1* gene mutation.

The patient received one cycle of paclitaxel (300 mg) and carboplatin (500 mg); however, carboplatin was switched to doxorubicin within 1 month due to poor tolerability as the patient developed generalized pain, rash, mucositis, and numbness. However, the same side effects occurred with doxorubicin; therefore, chemotherapy was stopped after only one additional cycle. Four months later, she was started on a sorafenib therapy trial (400 mg), but this was stopped within a week due to generalized body pain and fatigue. At this stage, CT with intravenous contrast was repeated and showed a stable tumor burden similar to that of CT prior to chemotherapy.

**FIGURE 1:**
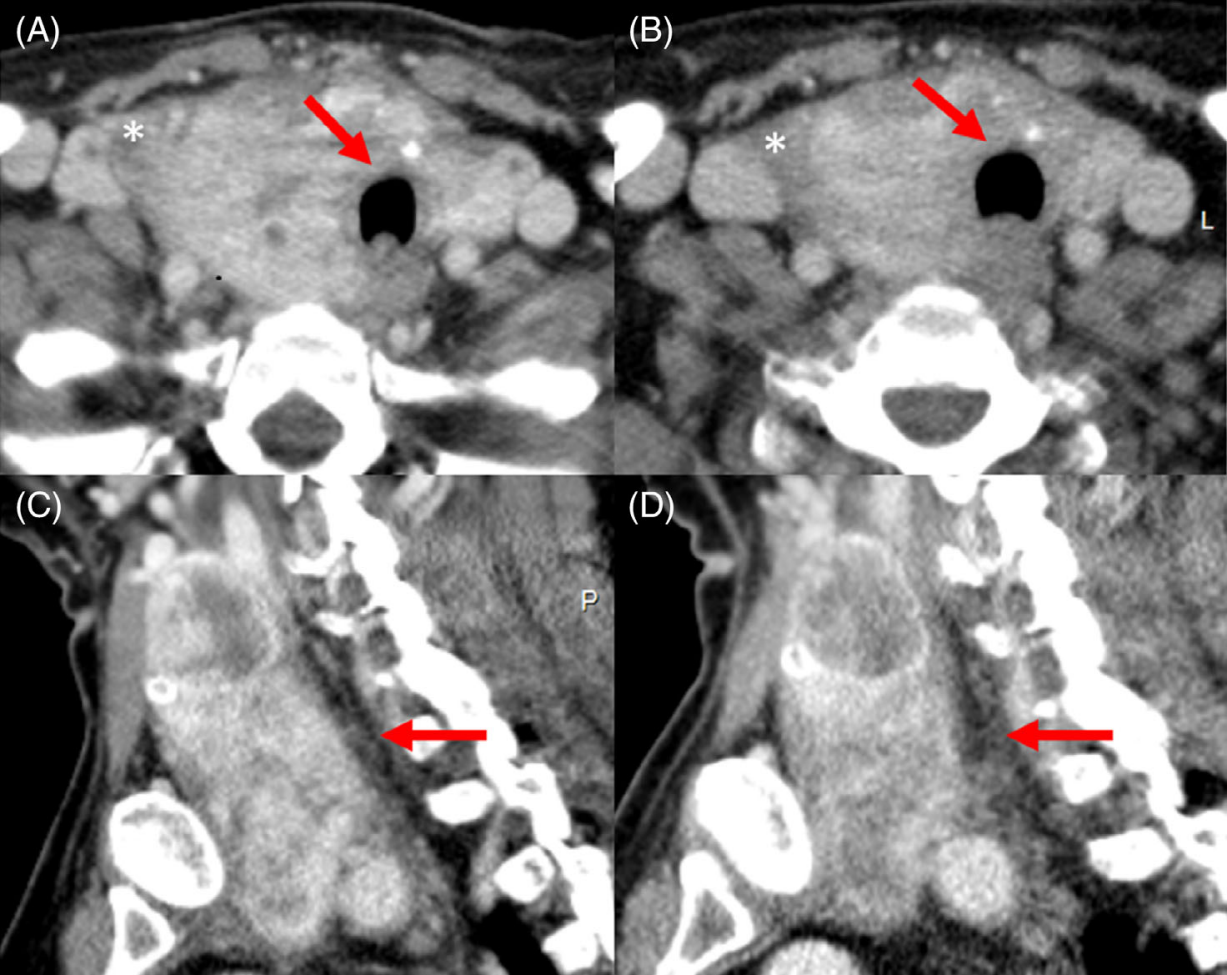
Axial and sagittal images from the CT examinations of the neck with contrast performed before and after Lenvatinib therapy. Axial (A) and sagittal (C) images before Lenvantinib treatment. Axial (B) and sagittal (D) images after Lenvatinib therapy. The axial images (A) and (B) demonstrate decreased mass effect on the trachea with widening of its lumen (arrows). Similar changes are noted on the right internal jugular vein (asterix). On the sagittal images (C) and (D), there is an overall subtle decrease in the size of the mass with decrease in the fullness of its posterior contour and increase in the fat posterior to the mass (arrow)

The patient was later commenced on lenvatinib 10 mg once daily, which she tolerated well with no significant side effects, except for fatigue. After 2 months of lenvatinib therapy, enhanced neck CT showed an interval decrease in the size of the mass with an interval reduction in the mass effect on the trachea. At this point, the tumor measured 7.2 × 6.6 × 3.7 cm (Figure 1B,D) compared to 9 × 9 × 4.4 cm before lenvatinib therapy (Figure 1A,C). The sternal lesion on the right internal jugular vein invasion was stable. The relationship of the mass to the trachea and esophagus remained the same. Following this regression in her disease, surgery was considered an option.

She underwent total thyroidectomy with central and right neck dissection after being taken off lenvatinib for 4 weeks. Intraoperatively, multiple adhesions were noticed around the thyroid tissues, which could be related to postradiation effects. There was a small residual area of internal jugular vein invasion; therefore, partial sidewall excision with primary closure was performed. No tracheal ring was removed as there was no gross invasion to the trachea or the esophagus visualized.

Postoperatively, her histopathology was again consistent with PDTC: 7 cm in the largest diameter, with positive margins and extensive vascular invasion. Gross ETE invaded the right jugular vein and strap muscles. Two perithyroidal lymph nodes were removed and tested negative for malignancy. Three months later, her thyroglobulin level was 2.95 ng/ml despite bone and lung metastasis, and a neck ultrasound revealed no residual or suspicious lymph nodes. Subsequently, she received 200 mCi of RAI therapy, and a posttherapy scan showed RAI uptake in the thyroid bed. Furthermore, there was an uptake in the lung and manubrium sterni in the same areas of the known metastases. Repeated neck, chest abdomen, and pelvis CT performed 6 months after lenvatinib therapy and within 3 weeks following RAI showed mild progression at the lung lesions with a stable bone lesion. She was then scheduled for a second RAI dose of 6 months.

## 3 DISCUSSION

**FIGURE 2:**
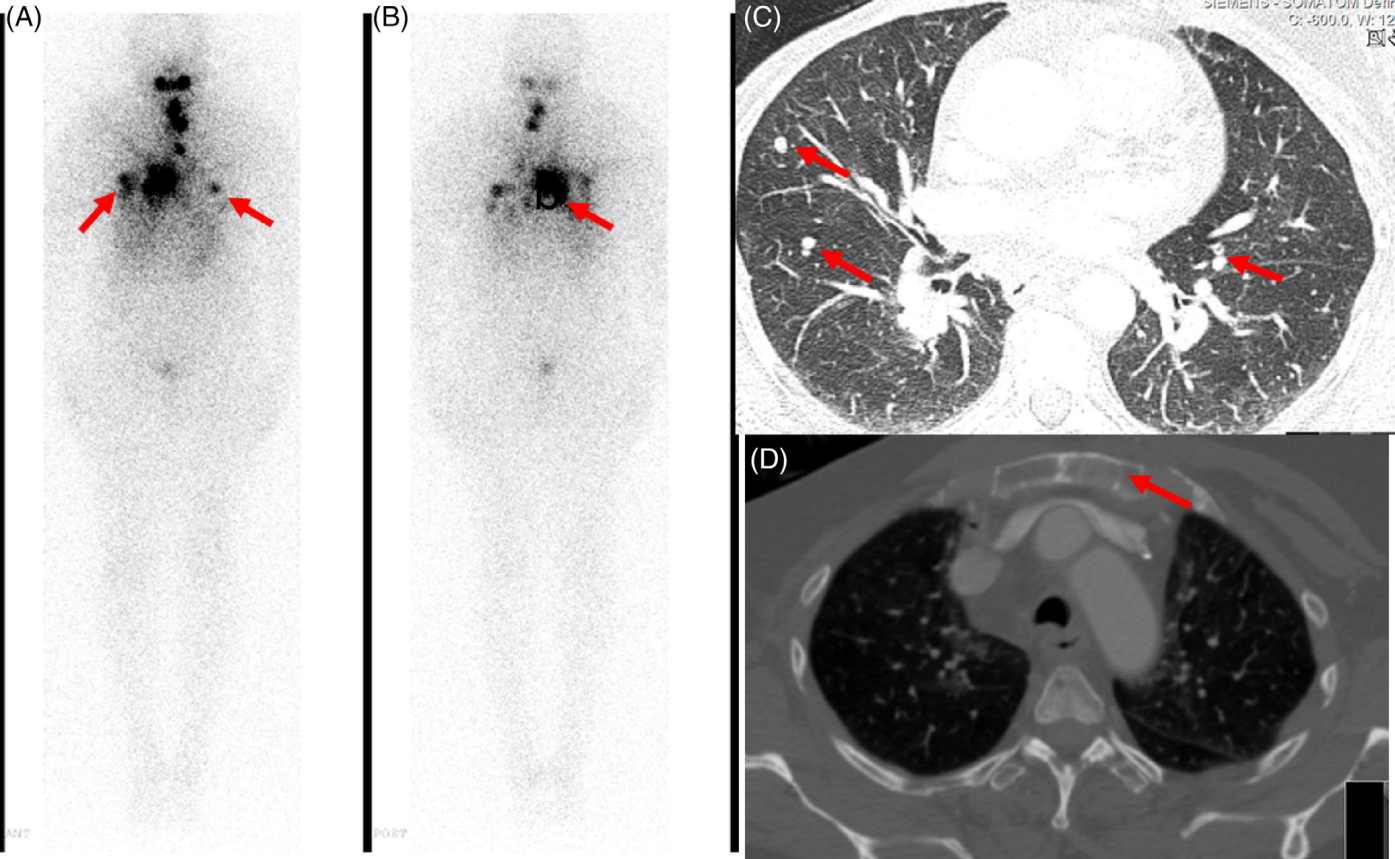
Iodine 131 nuclear study. Iodine 131 nuclear study (A) demonstrates the presence of multiple foci of abnormal tracer accumulation in the lungs (arrows) representing metastasis. (B) Increased tracer uptake in the sternum is also noted. (C) Pulmonary metastatic nodules were visualized on the CT chest (arrows). (D) A lytic sternum metastatic lesion (arrow) corresponds to the increased tracer uptake

In this case report, we demonstrated the potential of neoadjuvant TKI therapy for inoperable TC. Studies have shown that aggressive surgery in patients with TC exhibit gross ETE results in satisfactory locoregional control.^9^ However, in cases where surgeries with curative intent are not possible, neoadjuvant TKI therapy shows promising results. Initially, in our patient, lenvatinib was used as a palliative option, but it showed a therapeutic effect over the course of treatment (Figures 1 and 2). Lenvatinib targets vascular endothelial growth factor receptors 1‐3, fibroblast growth factor receptors 1‐4, RET, c‐kit, and platelet‐derived growth factor receptor α. Its antitumor activity is thought to be due to its antiangiogenic properties and direct antitumor effects.^10^

Lenvatinib and sorafenib have been reportedly used preoperatively in patients with differentiated TC and in those with PDTC (as in our patient) to shrink the tumor size in order to facilitate ensuing surgery and to spare the patient from the morbidities of a more aggressive resection possibly including laryngectomy, tracheostomy, and esophageal reconstruction. Table 1 presents a series of TC case reports that employed neoadjuvant TKI therapy and their subsequent outcomes.^11^, ^12^, ^13^, ^14^, ^15^, ^16^, ^17^, ^18^ Two of these cases had recurrent locally invasive TC,^12^, ^17^ and the other cases were newly diagnosed with advanced TC. The tumor size in the series ranged between 4 and 8 cm, with an effect that was observed after a short period of 8 weeks and as long as 98 weeks. The patients' ages ranged from 20 to 81 years. None of the patients had a T4b TNM staging, which is probably due to the serious consequences of developing a fistula as a side effect from TKI therapy, particularly lenvatinib therapy.^19^

**TABLE 1:** A series of TC case reports that utilized neoadjuvant TKI therapy and their subsequent outcomes

Study reference	Age (years)/Sex	TC histology	TNM	TKI^a^ (daily dose) EBR^b^ (+/−)	Duration (weeks)	Outcome
Gay et al^11^	81/F^c^	PDTC^d^	T4aNXM0	Lenvatinib (10 mg) EBR+	8	Lesion size was slightly reduced, and dysphonia improved.
Molinaro et al^12^	62/F	PDTC	T3bNxMx	Lenvatinib (10‐20 mg)	8	Reduction of tumor volume, and compressive effects of the cervical necrotic pathology. Patient died 1 month later secondary to pulmonary embolism.
Stewart et al^13^	73/F	PTC^e^	T4a N0 M1	Sorafenib (800 mg)	4	Reduction in tumor volume from 31 × 59 × 32 mm to 17 × 28 × 22 mm.
				Lenvatinib (14‐24 mg)	14	
Danilovic et al^14^	20/M^f^	PTC	T4aN1bM1	Sorafenib (800/day) EBR+	52	Reduction of the cervical mass, metastatic lymph nodes, and pulmonary nodules.
Tsuboi et al^15^	73/M	PTC	T4a N1b M0	Lenvatinib 14 mg	22	Tumor decreased in size by 84%, and the cervical lymph nodes by 56%.
Nava et al^16^	32/M	PTC	pT4a N1b Mx	Sorafenib (800 mg per day)	24	70% Tumor reduction.
Sukumar et al^17^	69/M	PTC	N/R	Lenvatinib (10‐20 mg)	71	Decreased size of paraesophageal mass from 9.3 × 4.3 × 7.1 cm to 2.6 × 1 cm.
				EBR+	4	Neck CT scan lacked evidence of residual or recurrent disease.
	69/F	PTC	N/R	Lenvatinib (4–24 mg)	98	
				EBR+	5	Decrease in the size of bilateral pulmonary nodules and left thyroid mass, and mediastinal lymph nodes. Persistent invasion of the trachea, vocal cord, and esophagus.
Iwasaki et al^18^	75/F	PTC	N/R	Lenvatinib (10‐14 mg)	16	Tumor shrank upon treatment, and the major axis was reduced from 68 to 48 mm in diameter.

Abbreviations: CT, computed tomography; EBR, external beam radiation; F, female; M, male; N/R, not reported; PDTC, poorly differentiated thyroid carcinoma; PTC, papillary thyroid carcinoma; TC, thyroid cancer; TKI, tyrosine kinase inhibitors.

^a^ Tyrosine kinase inhibitor.

^b^ External beam radiation.

^c^ Female.

^d^ Poorly differentiated thyroid cancer.

^e^ Papillary thyroid cancer.

^f^ Male.

Common adverse events in patients receiving lenvatinib include hypertension, asthenia, diarrhea, proteinuria, and decreased appetite, and these were reported in the present case series (Table 2). Fistula formation and mucosal perforation are among the most serious side effects of TKI therapy. One of the patients in the series developed a double esophageal perforation at approximately 21 cm from the nostrils (without a fistulous tract with the trachea) while receiving lenvatinib therapy.^13^ The tumor histological type, tumor extension, TKI type and duration, EBRT, and age could all potentially contribute to the development of esophageal perforation.

**TABLE 2:** Reported adverse events experienced by the patients in the case series

Study reference	Reported comorbidities	Reported adverse event
Gay et al^11^	HCV^a^ liver disease Atrial fibrillation PE^b^	Weight loss, fatigue, dermatitis, asthenia, mild hypertension, and mild dysphonia
Molinaro et al^12^	N/R	Double esophageal perforation; patient died from pulmonary embolism
Stewart et al^13^	N/R	Gastrointestinal side effects, significant QTc prolongation, weight loss, stomatitis, and diarrhea
Danilovic et al^14^	N/R	Fatigue, hand and foot skin reaction, and diarrhea
Tsuboi et al^15^	N/R	Grade‐III proteinuria and hypertension
Nava et al^16^	N/R	Hypertension and grade‐II hand‐foot syndrome
Sukumar et al^17^	N/R	Axillary abscess, myalgias, and dysgeusia
Iwasaki et al^18^	CLL^c^	Neuropathy, myalgias, dyspnea, and hypertension
Iwasaki et al^18^	N/R	Grade‐II HT, grade‐II hand–foot syndrome, grade‐II anorexia, and weight loss

Abbreviations: CLL, chronic lymphocytic leukemia; HCV, hepatitis C virus; HT, hypertension; N/R, not reported; PE, pulmonary embolism; QTc, corrected QT interval.

^a^ Hepatitis C virus.

^b^ Pulmonary embolism.

^c^ Chronic lymphocytic leukemia.

In this series, the favorable neoadjuvant effect of TKI was reported in both new and recurrent cases, regardless of whether they had received concomitant EBRT. In some patients, including the two patients reported by Sukumar et al,^17^ neoadjuvant lenvatinib also resulted in a significant quality of life benefit as it prevented the necessity for a laryngectomy procedure. The positive effect of neoadjuvant TKI was also noted in metastatic lesions, including metastatic lymph nodes and lung metastasis.^14^, ^17^ Our patient did not receive a repeated chest CT after the lenvatinib therapy period. Instead, it was performed 6 months later when mild progression of pulmonary metastasis was observed; therefore, it is difficult to judge the effect of lenvatinib on the lung lesions in our case.

## 4 CONCLUSION

In summary, we report a case of PDTC, initially considered inoperable, that was fully resected after preoperative lenvatinib treatment without any major perioperative complications. TKI therapy, such as lenvatinib, is a feasible option in patients with inoperable TC and can stabilize the lesion size or even reduce it, leading to a more favorable surgical outcome. In order to determine the maximum efficacy and outcome of neoadjuvant lenvatinib and sorafenib, further studies including larger samples are needed to investigate the optimal dosing and duration. Furthermore, it is necessary to determine the appropriate candidates and tumor factors for such a regimen.

## CONFLICT OF INTEREST

The authors have stated explicitly that there are no conflicts of interest in connection with this article.

## AUTHOR CONTRIBUTIONS

*Conceptualization; data curation; investigation; methodology; writing‐original draft*, K.A.; *Conceptualization; data curation; formal analysis; project administration; writing‐review & editing*, Y.A.; *Data curation; project administration; supervision; writing‐review & editing*, M.M.; *Formal analysis; investigation; project administration; resources; supervision; writing‐review & editing*, S.S.; *Conceptualization; validation; visualization*, R.D.; *Project administration; resources; supervision; writing‐review & editing*, H.M.

## ETHICS STATEMENT

For publication purpose, ethical approval has been taken and written informed consent was obtained from the patient.

## Data Availability

The data that support the findings of this study are available on request from the corresponding author. The data are not publicly available due to privacy or ethical restrictions.
